# The Neural Control Mechanisms of Gekkonid Adhesion Locomotion: The Effect of Spinal Cord Lesions

**DOI:** 10.3390/biomimetics7030098

**Published:** 2022-07-22

**Authors:** Xiaoqing Wang, Wenbo Wang, Zhendong Dai

**Affiliations:** Institute of Bio-Inspired Structure and Surface Engineering, College of Mechanical & Electrical Engineering, Nanjing University of Aeronautics & Astronautics, Nanjing 210016, China; wangxq1989@nuaa.edu.cn

**Keywords:** lizard *Gekko gecko*, adhesive system, locomotor performance, spinal cord lesion

## Abstract

**Objective:** the role of the supraspinal system in the neural control mechanisms of adhesion locomotor pattern formation was studied in lizard *Gekko gecko*. **Methods:** the locomotor performance and adaptation of the chronically lesioned *Gekko gecko* was documented before and after either partial or complete spinal lesions. They were filmed moving on a flat and smooth platform that was inclined at 0°, ±45°, and ±90°, as well as the horizontal mats and the vertical oak background board in the terraria, to evaluate locomotor functional recovery. The geckos were also tested on the platform by two half and nose-up or -down rotations in steps of 15° throughout 180° to investigate the recovery of the ability to respond dynamically to external perturbations. **Results:** after relatively small lesions of a hemisection, the locomotor performance was largely indistinguishable from that before and after a sham operation. During the initial period of recovery after the largest lesions of a dorsal or a ventral hemisection within 1 wk, the geckos behaved essentially as the complete spinal geckos, while permanent deficits in locomotor performance remained and did not decrease afterwards for ≥6 mth. **Conclusions:** by analyzing the correlation among locomotor performances, and between locomotor performances and spinal cord lesions, we suggest that the dorsal spinal pathways and ventral spinal pathways participate, respectively, in the control of the limb coupling, and in the deployment and the detachment of the adhesive apparatus. The present study will provide certain neurobiological guidance for the design of bio-robots, as well as sprawling robots inspired by the geckos.

## 1. Introduction

*Gekko gecko*, a large gekkonid lizard with a remarkable adhesion locomotion capability, make up an extremely diverse early diverging clade of lizards that are widely distributed in Asia and have been recognized as a valuable squamate species for studying the locomotor performance of pad-bearing geckos. Geckos can make locomotor strategies/patterns by changing their body and limb kinematics [1,2,3], i.e., with occurring limb modulation and by changing limb orientation, to adapt their efficient and stable locomotion to various terrain conditions. The specialized adhesive system of geckos further drives greater variation in the strategies for locomotion, e.g., geckos significantly alter their foot orientation during downhill locomotion, as well as the use of the Y-configuration on steep or inverted inclines [2,3]. One of the other modes of locomotor patterns could be altered quickly and become predominant according to the terrain. This allows the limbs to adjust to the requirements of maintaining locomotor stability. However, the neural control mechanisms of gekkonid adhesion locomotor pattern formation have not been widely studied, which seriously hampers the development of biorobots, as well as of sprawling robots inspired by the geckos.

Extensive spinal cord lesions have been widely applied in mammals [4,5,6] to reveal the contribution of different structures, i.e., spinal segments and supraspinal structures, to the normal control of locomotion by examining locomotor performance and adaptation, and these animals were able to recover a remarkably good locomotor capability after substantial damage to the partial spinal cord. In Reptilia, only turtles [7] and padless lizards [8,9,10,11] have shown anatomical and functional recovery after a complete transection in the lumbar or thoracic spinal cord, which leads to a partial and limited functional recovery of hind limb movements in these reptiles, including stepping locomotion and stepping–swimming movements in freshwater turtles, as well as in the mammals above.

However, in contrast to complete spinal lesions, the data on the effects of partial spinal lesions on locomotor function in sprawled quadrupeds are rather scarce, while previous studies have been mainly restricted to the regeneration of the reptilian tail [9,10]. In consideration of the presence of adhesive pads, which leads to significant differences in locomotor performance between the pad-bearing species and the padless species of lizards [2,12,13,14], we predict that their locomotor performance and adaptation after partial or complete spinal cord lesions may also be different.

Locomotor deficits caused by spinal cord lesions with different locations and degrees will be reflected in locomotor performance [4,6], which may also help to explain the correlation of different locomotor performances. Since the integrity of midlumbar segments was supposed to be crucial for the expression of spinal locomotion [5], the upper lumbar segment, between the third and the fourth lumbar vertebra level was selected for spinal cord lesions in the present study. The present study compared and characterized the impairments and subsequent recoveries of gekkonid locomotor performance with significant differences by quantitatively observing the significant deficits in the basic locomotor rhythm, the coupling between the contralateral fore and hind limb, and the ability to move on different chosen substrates that are associated with the microhabitat that the gecko occupies before and after a sham operation, as well as before and after either partial or complete spinal lesions. We also preliminarily investigated the recovery of the ability to respond dynamically to external perturbations, i.e., maintaining attachment and stability on a platform that periodic trapezoid tilted caused by periodic trapezoid tilting. These data were then analyzed to evaluate the locomotor functional recovery of the hind limbs in these animals.

Previous studies concerning the locomotion of geckos have placed a great deal of emphasis upon the process of adhesion and detachment, and the mechanism of setal adhesion with physical explanations proposed, i.e., frictional adhesion [13,15,16,17], while little emphasis has been put on the neural control mechanism of active adhesion and detachment. Since the effective deployment and detachment of the adhesive apparatus are closely related to the locomotor performance of the gecko on different substrates, we propose exploring this adhesion mechanism through the present study.

## 2. Materials and Methods

### 2.1. Surgical Procedures

Forty adult animals of both sexes, with a snout-to-vent length 155–171 mm and a body weight of 73.8–95.5 g, of the lizard species *Gekko gecko* (Junhao Wildlife Science & Technology Development Co., Ltd., Nanning, China) were used for the present study. All animals were maintained in terraria at 25–30 °C under natural light conditions that allowed the gecko to thermoregulate behaviorally. All the surgical procedures and experimental protocols were reviewed and approved by the forestry authorities of China and the Animal Care and Welfare Committee of our college. Efforts were made to minimize suffering and the number of animals used. Some of the experimental animals were used in the study over the same period [18].

After anesthesia with an intraperitoneal injection of 0.4% nembutal (0.75 mL/100 g body weight) over a period of several hours to achieve a surgical plane of anesthesia, which was defined by the total areflexia in response to nociceptive stimuli, an incision was made along the dorsal midline in the upper lumbar region. A laminectomy between the 3rd and the 4th lumbar vertebra levels was performed, and the dura was opened on the dorsum of the cord. Two types of partial spinal cord lesion—a dorsal (n = 12) or ventral (n = 12) hemisection—as well as a complete transaction (n = 8), and a sham operation (n = 8, received an operation that consisted of all of the procedures except spinal cord lesions) were performed on the geckos under a surgical operation microscope, by means of small scalpel, microsurgery forceps, and fine iridectomy scissors. For the ventral lesions, we used the technique mentioned by Lyalka et al. [6]. Surgically exposed neural tissue was bathed in mineral oil to keep the tissues of the wounded area from being directly exposed to the environment while forming the scab. Afterward, the incision was closed in anatomical layers. Post-operative antibiotics, a wound cicatrizing powder containing antibiotics, were given.

The geckos were then caged alone, and bupivacaine (0.25%, 1 mg/kg), a local anesthetic given for 5 min as a method of relieving post-operative pain, was given every 6–8 h for 24–48 h. Their locomotor recovery was followed and documented daily. They did not feed for at least three days after surgery, and then they were fed with live crickets (supplemented with vitamins) three times a week and were provided water ad libitum. They also needed help sloughing their skin, especially in the hind limbs and the tail.

### 2.2. Performance Tests

To analyze the locomotor performance and adaptation of the normal and lesionedgeckos, we made video recordings from the dorsal view at 30 frames/s using a digital camera and simultaneously recorded their motor behavior along with their foot position and toe attachment.

**Figure 1:**
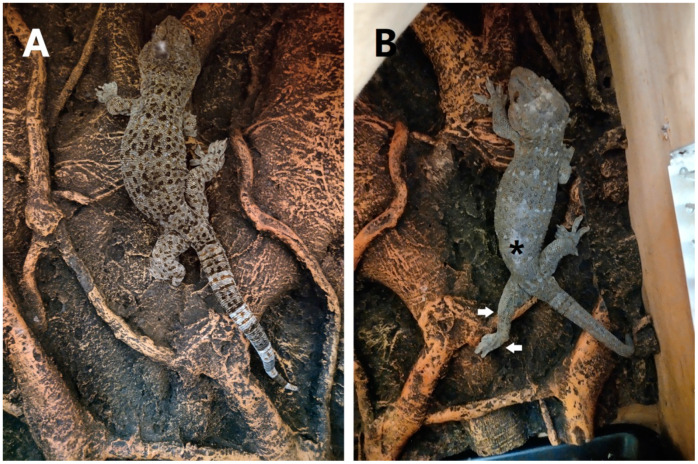
Examples of geckos on the vertical oak background board in the terraria before (**A**) and after (**B**) a dorsal hemisection (asterisk indicate the point of hemisection). (**A**) A normal gecko of alertly behaving in a resting position, the condition of foot position and toe attachment indicating that they actively load the adhesive system to resist gravity and respond dynamically to external perturbations. (**B**) A gecko after ≥6 mth post-hemisection, presenting a quite abnormal constrained locomotion since a permanently non-hyperextended state of the left hind limb caused the crus to come into line with the pes (arrowheads), while the right hind limb motioned well with a lower thoracic level since the need of grasping was the prominent part.

The geckos were filmed moving on a flat and smooth platform that was inclined at 0°, +45°, −45°, +90°, and −90°, and we caught the animal by hand prior to toppling. We also observed the locomotion of each gecko on the horizontal (coconut fiber) mats and the vertical oak background board in the terraria (Figure 1). The animals were encouraged to move at full speed by a gentle manipulation of the limbs and body with a thin paintbrush. Each animal had markers placed dorsally using white nail polish before the level running trials (smooth surfaces/mats), placed on the mid-pectoral/pelvic girdle and in-between the two girdle, as well digit III tips of the hind limbs mentioned by Birn-Jeffery and Higham [2,3]. We then measured the distance of the fore-aft axes (i.e., the coronal planes) between the two markers at the end of the swing phase, as well as at the beginning of the swing phase and termed it III_mid_. A positive III_mid_ represent the fact that the pes was placed anterior to the mid-pectoral marker, while a negative III_mid_ represent that the pes was placed posterior to the marker.

**Figure 2:**
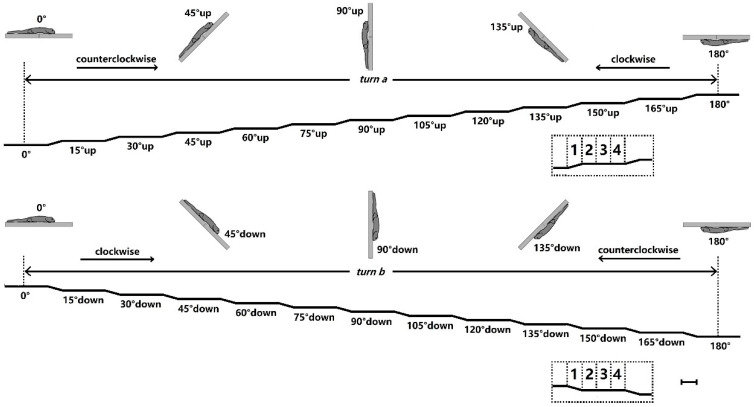
Experimental design for periodic trapezoid tilting. Angle of 0° corresponded to the normal (horizontal) orientation of the gecko with its dorsal side down, and rostral is to the right. Scale bar = 1 s for all. A total of 2 sequential half turns (turn a, nose-up rotations, counterclockwise, and the reverse; turn b, nose-down rotations, clockwise, and the reverse) were performed in 15° steps. Duration of each step was approximately 4 s, as the transition from one position to the next lasted approximately 1 s, and each position was maintained for approximately 3 s (insets).

The geckos were also tested on the platform by two half turns, nose-up (turn a) or -down (turn b) rotations, in the sagittal (pitch) plane by periodic trapezoid tilting in steps of 15° throughout 180° (inverted) (Figure 2). Before they were tested, since we discovered that the geckos became docile when the snouts were pinched, and that they were able to maintain a standing posture during perturbation tasks in the present study, a thin strip of adhesive bandage tape acted as a muzzle, as well as to prevent bites. The initial orientation of the animal was with its dorsal side down (0°). The transition from one position to the next lasted approximately 1 s, and each position was maintained for approximately 3 s. The angle of the fall was repeatedly tested in each gecko.

Muscle contractions after pinching their feet were assessed by visual inspection and palpation, since the electrode would lose contact along with molting, making it impossible to chronically implante electrodes for recording electromyographic (EMG) activity. The data were used to run ANOVAs to evaluate the adhesion locomotor recovery of the hind limbs in these animals.

### 2.3. Histological Procedures

At the termination of the experiment, the animals were killed with 0.4% Nembutal and were perfused transcardially with 0.75% physiological saline followed by 4% paraformaldehyde in 0.1 M-PB, pH 7.4. The position of each spinal cord lesion was checked visually by gross examination under a dissecting microscope. The region of the spinal cord damage was sectioned on a cryotome at 30 μm thickness in the transverse plane. The sections were then stained for Nissl substance with cresyl violet and were examined and photographed with an Olympus BX51 microscope equipped with an Olympus DP70 digital camera to verify the extent of the spinal lesions.

The damaged area in each type of partial lesion occupied approximately half of the spinal cord cross-section, while a few animals, with the extent of the lesion strongly differing from a hemisection after the observation of the magnified digital images of the sections, were excluded from the present study.

## 3. Results

### 3.1. Locomotor Performance in Intact Geckos

We first present the characteristics during unobstructed locomotion in the intact geckos. There were no significant changes in locomotor performance before and after a sham operation (n = 8, III_mid_ = −16.5 ± 2.3 mm, mean ± SD), while the latter could still be observed crawling autonomously on inverted surfaces in the terraria. In general four forms of locomotor patterns that exist in the gekkonid limbs depended on the inclination of the substrate during unobstructed locomotion. A comparison of locomotor performance and function in gekkonid limbs in specific environmental situations is shown in Table 1, where the descriptions of foot orientation were partially proposed and discussed by Birn-Jeffery and Higham [2,3] and Zhou-yi Wang et al. [1].

As was reported by Anthouy P. Russell [13] and Pesika et al. [15], strides of gekkonid fore limbs or hind limbs were generally symmetric and alternating when moving in a straight line. The gecko basically kept the two diagonal limbs in contact with the substrate during the level or 45°/90° upward locomotion, expressing an in-phase coupling of the diagonal limbs and a coupling of limb function (Table 1). When the two diagonal limbs were undergoing the phase of swing, the digits were carried through this phase of the locomotor cycle in a hyperextended condition [13]. Meanwhile, the center of gravity projected ventrally and was located along the line connecting the two supporting diagonal limbs [1]. Each pes was placed posteriorly and laterally to the ipsilateral manus with a lumbar level, and even with a lower thoracic level (i.e., a positive III_mid_) when moving on the vertical oak background board that needed to grasp the prominent part (Figure 1A). 

The hind limb digit trajectory was similar for the level and the 45°/90° upward locomotion. On the contrary, the kinematics of the hind limbs were altered to a greater extent than the fore limbs during the −45°/−90° downhill locomotion [3] (Table 1). The pes rotated posteriorly (opposite to the direction of locomotion) and the gecko held the ankle at a remarkably fixed angle during stance and swing, and the fore limb motion was only slightly adjusted, resulting in a functional de-coupling of the limb which indicated that the role of the hind limbs changed from driving to braking and/or stabilizing [2,3]. 

The animals were able to maintain attachment to a platform that was periodically trapezoid tilted. In particular, the geckos presented diagonally opposing feet during 90–180° nose-up rotations and the reverse in turn a, as well as during the 45–180° nose-down rotations and the reverse in turn b, showing the configuration of the ‘Y-configuration’, as was partly reported by Birn-Jeffery and Higham [3] and Z.-Y. Wang et al. [1]. Meanwhile, the abdomen of the geckos was observed to be detached at some distance from the platform on inverted inclines.

### 3.2. Effects of Complete Transection

Observations of the animals (n = 8) early after a complete transection on the horizontal mats and the vertical oak background board in the terraria showed that no voluntary activity of the hind limbs was observed. 

After recovered from anaesthesia, the geckos appeared to move around using their fore limbs only and dragged their hindquarters on the ground more or less immediately, with the heel of their hind limbs striking and the pes facing posteriorly. The hindquarters could not receive the support of the hind limbs when the geckos were threatening. With the alternating fore limb movements and the sinusoidal movements of the vertebral column, the center of gravity oscillated laterally and the geckos were able to lift and drop their hind limbs rigidity that initial complete paralyzed involuntarily, i.e., they had upward movements during swing and downward movements during stance. The gekkonid hind limbs exhibited an indistinct flexion-extension cycle during the stance phase and passive pendular movements during swing purely under the effect of gravity, and did not practically change limb orientation during locomotion, evidencing that their hind limbs could not be involved in pushing the body forward on a horizontal surface after a complete transaction. The dragged legs observed allowed the geckos to hook the horizontal mats with their claws, which would constrain locomotion.

These observed geckos moved their hind limbs with alternating movements of a small amplitude at a relatively slow walk for ≥8 wk, while the locomotor function to move the 90° upwards and the −45°/−90° downwards was almost completely abolished, and they practically did not recover during the whole observation period (≥3 mth) from the transaction. Meanwhile, the muscle activity of the hind limbs developed from unresponsive (III_mid_ = −97.3 ± 8 mm) to weak (III_mid_ = −85.5 ± 8.3 mm) when the feet were pinched.

The geckos were unable to maintain attachment to a platform that was periodically trapezoid tilted during the 105–180° nose-up rotations and the reverse in turn a, as well as during the 60–180° nose-down rotations and the reverse in turn b. In particular, the hind limbs were observed to be detached from the platform during the 90–105° nose-up rotation, as well as during the 105° sustained position in turn a, with only the attachment of the fore limbs. The geckos then dropped from the platform during the 105–120° nose-up rotations, coinciding with the critical angle of detachment in the toes of live geckos [16].

### 3.3. Effects of Dorsal Hemisection

**Figure 3:**
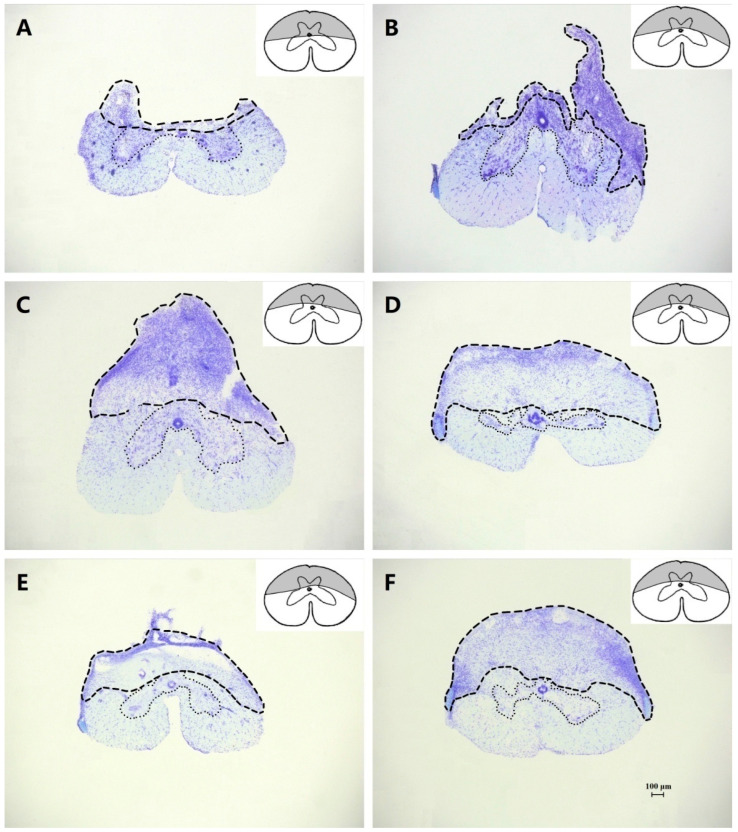
Photomicrographs and the corresponding schematic drawings of transverse sections, showing the extent of the spinal cord damage with dorsal lesions in individual geckos. Scale bar = 100 μm for all. In the photomicrographs, the lesioned area is delimited by broken lines, and the intact gray matter by dotted lines, with the remainder corresponding to the intact white matter. In the schematic drawings, the damaged area is shaded.

Most of the geckos (9 of the 12 lesioned) performed abnormal constrained locomotion. During the initial period of recovery after a dorsal hemisection within 1 wk, four geckos (the extent of the spinal cord damage of the individual geckos is shown in Figure 3A,B) behaved essentially as complete spinal geckos (III_mid_ = −94.8 ± 7.1 mm), and five geckos (the extent of the spinal cord damage of the individual geckos is shown in Figure 3C–E) had asymmetric hind limb locomotor movements when moving on the level mats. These 5 geckos observed a de-coupling of limb function between one fore limb and the contralateral hind limb since the pes rotated posteriorly and presented as a late complete transaction (III_mid_ = −83.1 ± 8.3 mm), while the contralateral fore limb and the hind limb were coupling as normal (III_mid_ = −17.4 ± 3.2 mm).

Subsequently, the hind limbs of the four geckos demonstrated paw drag with alternating movements of a small amplitude on the level mats (III_mid_ = −73.5 ± 6.7 mm), whereas, somewhat unusually, forward progress was severely hampered on the other substrates, since the digits of the pes were flattened onto the surface and could not either rotate preaxially nor lose contact with the surface by raising the distal end off the substrate, i.e., hyperextending the toes. This deficit caused the crus to come into line with the pes. The five geckos also presented constrained locomotion since they had a permanently non-hyperextended state of the disabled hind limb, although the contralateral hind limb motioned well during stance and swing (Figure 1B). This deficit did not decrease afterwards for ≥6 mth. 

The geckos were able to maintain attachment to a platform that was periodically trapezoid tilted. However, since the crus maintained to come into line with the pes, the abdomen of the geckos was observed to be kept close to the platform during the rotations, indicating a maximum inward pull, quite different from the intact ones.

The performance of the other three geckos remained thereafter good and had no significant differences in locomotor performance form the intact geckos (III_mid_ = −20.3 ± 3.5 mm). Histological analysis showed that there was an incomplete dorsal lesion (the extent of the spinal cord damage of the individual gecko is shown in Figure 3F).

### 3.4. Effects of Ventral Hemisection

**Figure 4:**
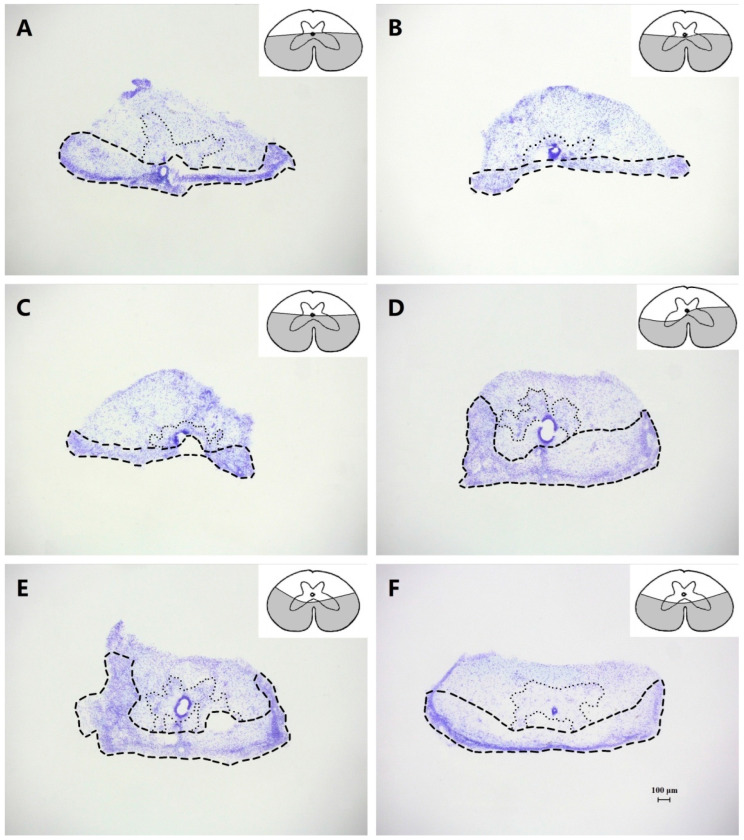
Photomicrographs and the corresponding schematic drawings of transverse sections, showing the extent of the spinal cord damage with ventral lesions in individual geckos. Scale bar = 100 μm for all. In the photomicrographs, the lesioned area is delimited by broken lines, and the intact gray matter by dotted lines, with the remainder corresponding to the intact white matter. In the schematic drawings, the damaged area is shaded.

There was also an initial period of recovery after a ventral hemisection within more or less 1 wk, and 5 of 12 geckos (the extent of the spinal cord damage of the individual gecko is shown in Figure 4A–C) behaved essentially as the complete spinal geckos (III_mid_ = −89.8 ± 8.5 mm). 

The five geckos then had symmetric hind limb locomotor movements without constraining locomotion when moving on the level substrate (III_mid_ = −63.2 ± 6.9 mm); however, there was a deficit in the fore- and hind limb coupling. The crus of the hind limbs sloped and faced laterally and slightly posteriorly, and faced posterolaterally. Since the first digit extended anterolaterally, the second to fourth digits extended posterolaterally, and the fifth pointed posteroventrally, the pes had an approximately posterolateral orientation. The pes was also placed below the lumbar and sacral level during quadrupedal locomotion. Meanwhile, the muscle activity of the hind limb developed from fair to vigorous during 1–2 wk postlesion when their feet were pinched. Eventually, the five geckos were capable of voluntarily climbing up and down with partial functional recovery, which was quite different from the geckos with the largest lesions of a dorsal hemisection, despite a limited ability to maintain locomotor stability on the vertical oak background board in the terraria.

The other seven geckos (the extent of the spinal cord damage of the individual geckos is shown in Figure 4D–F) had symmetric hind limb locomotor movements when moving on the level substrate without an initial period of recovery (III_mid_ = −49.4 ± 5.2 mm), while the pes had an abnormal lateral orientation, and was placed at a lumbar and sacral level, suggesting a considerable change in the locomotor performance of the hind limbs before and after the lesions. The seven geckos regained the capability to move on the other substrates after a few days of the procedure, both uphill and downhill. 

The five geckos were unable to maintain attachment to the substrate that was periodically trapezoid tilted during the 120–180° nose-up rotations and the reverse in turn a, as well as during the 90–180° nose-down rotations and the reverse in turn b. Meanwhile, the seven geckos were unable to maintain attachment during the 135–180° nose-up rotations and the reverse in turn a, as well as during the 105–180° nose-down rotations and the reverse in turn b. The fore limbs and hind limbs seemed detached simultaneously from the platform during the 120–135° (for the five geckos) and the 135–150° (for the seven geckos) nose-up rotations in turn a, as well as during the 105–120° nose-down rotations in turn b, differing with the complete spinal ones. Since the diagonal feet still presented approximately opposite directions, the failure of the geckos to provide a sufficient inward pull after the lesions was the reason for detachment.

**Figure 5:**
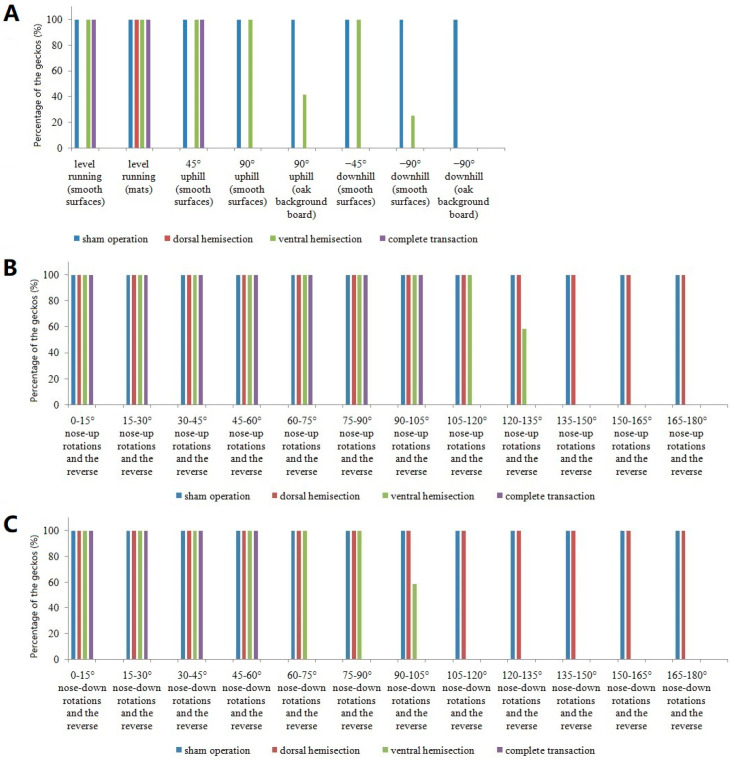
Comparison of persistent adhesion locomotor deficits in the geckos before (i.e., a sham operation (n = 8)) and after chronic partial (i.e., a dorsal hemisection (n = 12) or a ventral hemisection (n = 12)) or complete (i.e., complete transaction (n = 8)) spinal cord lesions. (**A**) Comparison of the percentage of the geckos (%) with 0°, +45°, −45°, +90°, and −90° adhesion locomotion on a flat and smooth platform, as well as the horizontal (coconut fiber) mats and the vertical oak background board in the terraria. (**B**) Comparison of the percentage of the geckos (%) tested on the platform by nose-up (turn a) rotations in steps of 15° throughout 180°. (**C**) Comparison of the number of the geckos tested on the platform by nose-down (turn b) rotations in steps of 15° throughout 180°.

A comparison of the persistent locomotor deficits in the geckos after chronic partial or complete spinal cord lesions is shown in Table 2 and Figure 5.

## 4. Discussion

The present study indicated that the gekkonid adhesion locomotor deficits, mainly deficits in basic locomotor rhythm, limb coupling, and locomotor stability (to respond dynamically to external perturbations), largely depended on the degree of the spinal cord impairments, and the time of observation after the lesion.

### 4.1. Maintenance of Locomotor Stability

**Figure 6:**
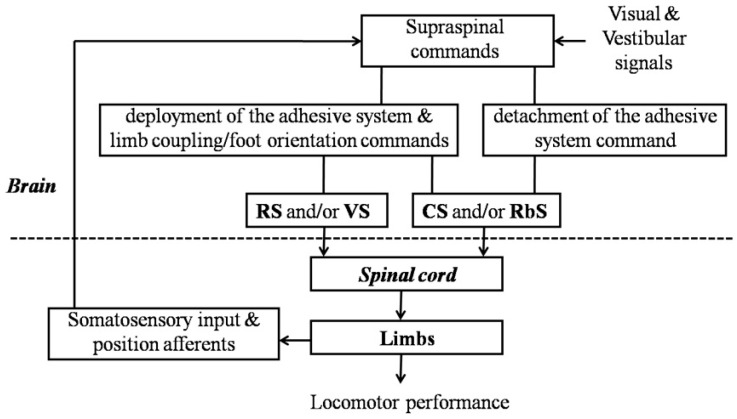
Main components of neural control mechanisms of adhesion locomotor pattern formation in pad-bearing species of lizards. Spinal cord generates the basic locomotor rhythm, and the effect is not being superimposed onto the effects of supraspinal commands, which are generated on the basis of sensory information and transmitted by the major descending tracts (reticulospinal (RS), vestibulospinal (VS), corticospinal (CS), and rubrospinal (RbS) tracts). Dorsal spinal pathways (CS and/or RbS) and ventral spinal pathways (RS and/or VS) participate, respectively, in the control of the deployment of the adhesive apparatus and the limb coupling/foot orientation. Meanwhile, dorsal spinal pathways (CS and/or RbS) participate in the control of the detachment of the adhesive system.

The maintenance of locomotor stability on different substrates involves the correlation among adhesion locomotor performances. In the intact geckos with accepted visual and vestibular signals, as well as somatosensory inputs and position afferents (Figure 6), the hind limbs could be used to maintain lateral stability and to compensate for dealing with the forward toppling moment when moving on steep declines with the deployment of the adhesive system and the concomitant clinging [3]. They would participate in overcoming gravity and maintaining the specified stability by pulling inward with both feet when climbing on a vertical surface [15]. Meanwhile, with the aid of the configuration of the ‘Y-configuration’ on steep or inverted inclines [2,3], the geckos used diagonally opposing feet that pull inward for maximum stability [16], greatly increasing the ability to respond dynamically to external perturbations. The lesioned geckos that behaved essentially as the complete spinal geckos, including some geckos with a dorsal (Figure 3A,B) or ventral (Figure 4A,B) hemisection with an initial period of recovery within 1 wk, had deficits in locomotion in some specific environmental situations. In particular, the hindquarters oriented on one side or the other and toppled during −45° downhill locomotion, which illustrated the medio-lateral and fore-aft imbalance.

After relatively small lesions of a hemisection (Figure 3F), the locomotor performance of the geckos was largely indistinguishable from that of the pre-lesion period control, for both uphill and downhill, especially for the maintenance of locomotor stability. We suggest that the geckos had the ability, to some degree to, participate in the locomotor compensation observed for the lesions, since the interruption of the supraspinal structures was generally less severe. Since the cortico- and rubrospinal tracts are complementary in the control of locomotion and other motor behaviors [4,19], their little effect on locomotor performance after relatively small lesions of a dorsal hemisection was not surprising, although whether the fibers that originate from the telencephalon reach the lower spinal cord in the reptilians are uncertain [20,21].

Since in the geckos with the largest lesions of a dorsal (Figure 3A,B) or ventral (Figure 4A–C) hemisection, there were permanent deficits in locomotor performance throughout the testing period for ≥6 mth that were reflected in the limb coupling, the position of the hind limbs in relation to the trunk, and the maintaince of locomotor stability, the remaining descending pathways were insufficient to completely compensate for the deficits caused by the lesions. Although the fore- and hind limb coupling was inconsistent, and the hind limbs might not have produced an impulse that generated work whilst climbing up along with less efficiency during locomotion, the geckos with the largest lesions of a ventral hemisection were still capable of voluntary uphill and downhill locomotion that gradually restored, eventually. We suggest that since the adhesive pads of the hind limbs with a recovered normal function provided enough shear adhesive force to counteract the lateral movements of the center of gravity [2] when going the 90° uphill or the −45°/−90° downhill, it was sufficient for maintaining the stability and security of locomotion and avoiding triggering catastrophic overturning albeit with an absence of the functional coupling of the limbs. However, the hind limbs of these lesioned geckos might have been unable to provide a sufficient inward pull for maximum stability dynamically, which was demonstrated by the inability to maintain attachment to a platform that was periodically trapezoid tilted during the 120–180° nose-up rotations and the reverse in turn a, as well as during the 90–180° nose-down rotations and the reverse in turn b.

During locomotion, the basic locomotor rhythm is capable of determining many of the underlying structures of the step cycle, since the descending signals and spinal mechanisms, which are able to ensure each period of the increased activity acts only on the appropriate muscles, are being superimposed onto the basic locomotor rhythm [22]. In geckos, the functional coupling of the limb and the stability and security of motion could not be simply superimposed onto the recovery of the basic locomotor rhythm, as well as the phasic modulations of the level of excitability of the spinal interneurons. The present study suggests that the former two locomotor performances were under the control of the other nervous system, probably the supraspinal structures, and the dorsal and ventral pathways participate, respectively, in the expression of locomotor performances. In geckos, the stability of locomotion may not be related to the coupling of the limbs, which is reflected in downhill locomotion and in various complex terrains, since the locomotor strategy of the front limb propulsion and hind limb stabilization reduces gekkonid maneuverability but improves the stability [2,3]. The locomotor strategy that the coupling of the fore limb and hind limb is inconsistent but does not affect locomotor stability on different substrates has been reflected in the design of a sprawling robot, for both uphill and downhill locomotion [23].

Meanwhile, asymmetric hind limb locomotor movements for the geckos with a dorsal hemisection (Figure 3C–E) when moving on the level mats, which did not seem to be a deficit in the geckos with a ventral hemisection, might indicate that there was a behavioral expression of the change in the intra- and interlimb coordination. In order to verify this mechanism, an examination of the adhesion locomotor performance of the geckos after more extensive lesions need to be evaluated further, i.e., after only one ventral/dorsal quadrant is spared (to lesion a 3/4 section of the spinal cord between the third and the fourth lumbar vertebra).

### 4.2. Neural Control of the Deployment and the Detachment of the Adhesive System

Of significance in our observations was the fact that the geckos with a complete spinal lesion were unable to actively load the adhesive apparatus, based on the inability to move the 90° uphill and the −45°/−90° downhill, as well as the failure of the hind limbs to attach to a platform that was periodically trapezoid tilted during the 90–105° nose-up rotation, and during the 105° sustained position in turn a in the present study. Meanwhile, the animals with partial spinal lesions, even with lesions leaving only the dorsal or ventral pathway, could provide effective deployment, respectively. Russell and Higham [24] concluded that the geckos slowed down on inclines of 10° and greater compared to level locomotion; however, the padless species did not, since an additional level of control during the deployment and detachment of the adhesive system was required. As the stability and security of gekkonid motion are directly associated with the adhesive system and permit the gecko a wide variety of substrates to be traversed [13], we suggest that normal geckos generate a locomotor pattern of controllable attachment and release that depend on supraspinal inputs, which are not being superimposed onto the coupling of the limb. Birn-Jeffery and Higham [2,3] reported that it will not affect the lack of change in the timing of deploying and detaching the gekkonid adhesive system on different terrain, whether the limb is coupling or de-coupling. Here, since the complete spinal geckos were unable to actively load the adhesive apparatus neither upward nor downward locomotion, the supraspinal structures, which are supposed being superimposed onto the spinal mechanisms, rather than the perception of gravitational effects, are prerequisite for the deployment of the clinging apparatus, which is somewhat different from the conclusion from Russell and Higham [24].

The investigation of the functional analysis of the gekkonid foot by Anthouy P. Russell [13] revealed that flexor tension is a requirement for successful adhesion. With vigorous muscle activity when the feet were pinched, intact geckos or those with relatively small lesions of a hemisection were able to maintain the flexor tension of the hind limbs. Observations of the animals with the largest lesions of a dorsal or ventral hemisection indicated that the remaining descending pathways were insufficient to completely compensate for the deficits caused by the lesions due to the de-coupling of the limb when moving on unobstructed level or the 45°/90° upward surfaces; however, effective adhesion can be assured rapidly. These observations allow us to conclude that the dorsal spinal pathways and ventral spinal pathways participate, respectively, in maintaining the flexor tension of the hind limbs to some extent and therefore are in the control of the deployment of the adhesive apparatus.

The gecko completely removes the adhesive system from the substrate under strict control by hyperextending its toes [13], otherwise the delicate subdigital setae cannot be protected from damage and the locomotion will be constrained. We suggest that when intact or hemisection lesioned geckos were alertly behaving before moving and had a resting position before the beginning of the propulsive phase, they actively loaded the adhesive system to respond dynamically to external perturbations. However, since the complete spinal geckos could not actively deploy the clinging apparatus, the subdigital pads of the hind limbs were held in a permanently hyperextended configuration involuntarily. In fact, the videography also revealed that the configuration of the pes in complete spinal geckos did not seem to change much throughout the locomotor cycle on different terrain. With unresponsive-to-weak muscle activity when the feet were pinched, the geckos with a complete transaction or the early largest lesions of a dorsal or ventral hemisection might have been unable to maintain the flexor tension of the hind limbs, which resulted in the relaxing of the flexor muscles in the hind limbs and feet. Then the pull on the lateral digital tendons was negated and the dorsal interossei contracted, which resulted in the release of the digits by means of hyperextension [13].

The unusual observation of the gecko with the largest lesion of a dorsal hemisection on the level mats and smooth surfaces revealed that dragging paws and non-detachment of the pes occurred simultaneously. They kept their digits in a non-hyperextended position and could not actively detach the clinging apparatus when moving on the non-mat terrain. Since geckos possess directional adhesion which is along the long axis of the digit [17,25], and the shear adhesive force of the non-hyperextended pes increased with the pulling force provided by the fore limbs, the crus came into line with the pes and the digit angle decreased passively, indicating a maximum inward pull. These gekkonid hind limb locomotor performances were similar to the normal ones observed during downhill locomotion; however, the subdigital setae might not have been protected. Subsequently, the digits remained in a constant position and the gecko could not locomote anymore. Stewart and Higham [25] proposed that the main regulatory mechanism of a gecko for reducing adhesive force is by the active hyperextension of the toe. On the contrary, since lizards of the padless species with paw drag of the hind limbs after spinal cord lesions do not perform constrained locomotion [8,9,10], different adaptive requirements of the exquisite control of the toe are present between pad-bearing species and padless species.

We suggest that in the geckos with a dorsal hemisection, the lesion to the spinal cord was irregular (Figure 3C–E) and resulted in asymmetric hind limb locomotor movements on the horizontal mats after the procedure, while forward progress was severely hampered on the other substrates, which was also a strong indication that there was a behavioral expression of the change mainly in the intralimb coordination. Rossignol et al. [4] proposed that changes in intralimb coordination are probably responsible for the paw drag in animals with a large lesion of the dorsolateral funiculi. The side with normal locomotion of the hind limbs spared some dorsal pathways, and the remaining pathways were sufficient to completely compensate for the locomotor deficits of this side caused by the lesion, while they were insufficient to compensate for the normal locomotion of the contralateral hind limb, however. In the side with deficits in locomotor performance, including paw drag and a permanent non-hyperextension of the digits, there might have been a complete transected of dorsal pathways, interrupting both the cortico- and rubrospinal tracts, and the remaining pathways, the ventral pathways, were insufficient to compensate for the locomotor deficits. The present study indicated that dorsal spinal pathways are of crucial importance for the detachment of the adhesive system. When a part of these pathways is spared, the detachment of the clinging apparatus can be restored rapidly, as well as locomotor abilities on different terrain, while the remaining descending pathways are capable of producing the requisite locomotor adaptations.

### 4.3. Correlation Betweensupraspinal Structure and Adhesion Locomotor Performance

In geckos, supraspinal structures might participate in the coordinated control, trade-offs, and development of compensatory strategies of adhesion locomotor performance to ensure locomotor ability on different terrains, including the relevant muscular recruitment related to the coupling of limbs and the production of requisite inward pull, the control of the attachment and detachment of the adhesive apparatus, and the regulation of the fixed time of deployment and disengagement of the subdigital pads, by transmitting neural commands to spinal neurons, and via them to limb muscles (Figure 6). The accurate control of the supraspinal pathways reflects a trade-off between maneuverability and stability.

The effects of microstimulation [26] and chronic lesions [4] indicated that the pontomedullary reticular formation pathways are closely correlated with interlimb coupled responses in fore limbs and hind limbs, lateral stability, and weight support. Since damage to the ventral spinal cord caused dramatict impairment in the locomotion in cats, as well as in rabbits, Lyalka et al. [6] suggested that reticulospinal and vestibulospinal pathways are important for stepping. These ventral pathways were supposed to activate the spinal controllers and provide the necessary muscle tone [27], while a high tonus in the extensor muscles is also a necessary condition for supporting the body during standing [28], which explains why the geckos with a complete spinal lesion could not support their body with their hind limbs. Meanwhile, since lesions of the red nucleus leads to evident but relatively mild locomotor deficits during unobstructed locomotion, mainly performed to move with an asymmetric gait persistently [29], with the help of unit recording and microstimulation studies, Lavoie and Drew [22] suggested that the rubral neurons can contribute to the normal control of walking by the widespread influence of the activity of the physiological flexor and extensor muscles of the limbs. The asymmetric gait in rats after lesions of the red nucleus [29], combined with asymmetric hind limb locomotor movements in geckos with a dorsal hemisection, also indicated that unilateral lesions of either the red nucleus or the rubrospinal pathway lead to persistent deficits of intra- and interlimb coordination, since the lesions may influence both the flexor and extensor motoneurons. As a result, the pes is incapable of rotating preaxially since the pronator profundus may not contract, which leads to the most anterior position of the foot being unable to be increased with paw drag.

Our discussion emphasizes the well-developed rubrospinal pathway, since reptiles lack a motor cortex [20,21], and the vestibulospinal tract was considered not well developed [8]. We regard the active procedure of gekkonid digital attachment and detachment as the extraordinary grasping and releasing of the substrate, respectively. Kan and McCurdy [30,31] investigated the role of the magnocellular red nucleus of monkeys in controlling reach-to-grasp movements related to single-unit discharge and kinematic data from the same individual trials of the whole-hand. They observed that there were strong correlations between the discharges of the individual rubral neurons and the parameter of the corresponding metacarpi-phalangeal extensions. They also proposed that the distal muscles of the limbs were most frequently correlated with rubral discharge, while the extensors were more frequently correlated than the flexors. Wen-bo Wang et al. [32] in our college induced stable motor behaviors by means of microelectrode implantation technology into the midbrain of *Gekko gecko*, to study the central mechanisms in motor control. Their unpublished observations showed that the electrical stimulation of the red nucleus resulted in a single movement of digital hyperextension in the contralateral hind limb, as well as non-hyperextension. The latter induced a pause in forward progress. We suggest that in the gecko, the red nucleus participates in the exquisite control of the toe, especially in the control of the active detachment of the adhesive apparatus by sending neural commands, which are transmitted in the rubrospinal tract, to spinal neurons, and via them to the distal muscle and tendon systems, especially the distal extensors. Meanwhile, when the rubrospinal pathway is completely damaged, the remaining descending pathways are incapable of compensating, and it is difficult to recover digital hyperextension and severely hampers forward progress.

This paper has provided the background necessary for a thorough investigation of neural control mechanisms of gekkonid locomotion, mainly the spinal and supraspinal systems, and led to a greater understanding of the adhesive process. We also actively provided a reliable and stable subject under extremely controlled conditions with permanent non-hyperextension, accompanied by the production of a maximum inward pull not observed in the recently euthanized geckos, since the digits of the pes of the latter needed to be manually depressed onto the surface [25]. With this subject, it might be possible to verify whether geckos can achieve rapid escape movements by simply jumping autonomously when climbing vertical surfaces, or efficient and stable locomotion purely under the effect of gravity when inverted on the ceiling, without hyperextending their toes. It may suppose the requirement to reduce inward pull by manually altering foot orientation, as well as changing the position of the hind limbs in relation to the trunk. Moreover, a further study of the correlation between the dynamic response to locomotor perturbations and the rotational speed of a platform is considered, which also reflects a trade-off between maneuverability and stability. We added a sentence to the last paragraph that “We will further investigate the connections to the adhesion locomotor effects made by the supraspinal system, by stimulating within the supraspinal structures (particularly the red nucleus) using brief trains of electrical pulses, by quantifying the electrical activity related to the limb muscle contractions and averaging the tiny fluctuations with respect to the time of the stimulus pulses (stimulus-triggered averaging) [33], which will provide certain neurobiological guidance for upper-level programming in control framework of bio-robots, as well as sprawling robots inspired by the geckos.

## Figures and Tables

**Table 1:** Comparison of locomotor performance and function in gekkonid limbs at different inclines.

Inclines	Fore Limbs		Hind Limbs	
	Locomotor Performance/Foot Orientation	Locomotor Function	Locomotor Performance/Foot Orientation	Locomotor Function
level	extend anterolaterally	brake	extend laterally	driver/propulsion
45°/90° uphill	extend forward and upward	propulsion	extend laterally and anterolaterally	driver
−45°/−90° downhill	extend anteriorly and anterolaterally	propulsion	rotate posteriorly and posterolaterally	brake and/or stabilizer
inverted (180°) surface	extend anterolaterally	propulsion	extend posterolaterally	brake and stabilizer

**Table 2:** Comparison of persistent adhesion locomotor deficits in the geckos after chronic partial or complete spinal cord lesions.

Inclines	Complete Transaction (n = 8)	Dorsal Hemisection (n = 12)	Ventral Hemisection (n = 12)
level locomotion (smooth surfaces)	Yes (paw drag with weak alternating movements)	No (paw drag with digital non-hyperextension)	Yes (paw drag with digital hyperextension)
level locomotion (mats)	Yes (paw drag with weak alternating movements)	Yes (paw drag)	Yes (paw drag)
45° uphill (smooth surfaces)	Yes	No	Yes
90° uphill (smooth surfaces)	No	No	Yes
90° uphill (oak background board)	No	No	Poor
−45° downhill (smooth surfaces)	No	No	Yes
−90° downhill (smooth surfaces)	No	No	Poor
−90° downhill (oak background board)	No	No	No
periodic trapezoid tilting	maintain attachment during the 0–105° nose-up rotations and the reverse in turn a, as well as the 0–60° nose-down rotations and the reverse in turn b	Yes (maintain attachment in turn a, as well as in turn b)	maintain attachment during the 0–120° (n = 5) and the 0–135° (n = 7) nose-up rotations and the reverse in turn a, as well as the 0–90° (n = 5) and the 0–105° (n = 7) nose-down rotations and the reverse in turn b

## Data Availability

The data used to support the findings of this study are available from the corresponding author upon request.
